# Double Rachitome for Opening the Spinal Canal

**Published:** 1830-10-01

**Authors:** 


### Double Rachitome, for opening the Spinal Canal.

The difficulties, or we should rather say delays, experienced in laying bare the medulla spinalis are familiar to us. The consequence is that we are less acquainted with the morbid conditions of this essential portion of the human body, than with those of any other part or parts of it. Our French brethren have laudably attempted to facilitate the operation by the invention of several instruments. We have seen one rachitome in this country; it consists of a sort of very strong chisel, curved in a semi-elliptical form, without any handle standing perpendicular to the blade. The back is very broad, and the mode of using it is to force it through the spinal arches on each side of the spinous ridge by blows of the hammer. We never used it nor saw it used, but we think the common mode by the saw and chisel must be preferable. We find that a M. Tarral has presented to the Royal Academy of Medicine what he calls a "double rachitome," by means of which the vertebral canal can be opened on both sides at once. Each branch of the instrument has a rest, arrêt, behind, to prevent the bone being forced down upon the medulla, which not unfrequently occurs; the branches are placed at a distance of eight lines from each other, a sufficient distance to prevent the medulla being injured in any part of its course; on the handle is a hook to raise the depressed pieces of bone, and the handle itself is placed obliquely with respect to the cutting edge to prevent any injury to the fingers of the operator. The two blades or branches are prevented from slipping by the spinous processes, which fix the instrument. Upwards of twenty spinal columns have been opened by the instrument, always in a very few minutes, and without any injury to the medulla.

Would the size of this instrument be adapted for inspecting children? At all events that would not signify greatly, as the spine in such subjects is examined without difficulty. We think that our own morbid anatomists would do well to procure this instrument, or invent a better if they can.

